# Microbial Production and Biomedical Applications of Lovastatin

**DOI:** 10.4103/0250-474X.49087

**Published:** 2008

**Authors:** A. Seenivasan, S. Subhagar, R. Aravindan, T. Viruthagiri

**Affiliations:** Biochemical Engineering Laboratory, Department of Chemical Engineering, Annamalai University, Annamalai Nagar-608 002, India

**Keywords:** Lovastatin, HMG-CoA reductase, low density lipoprotein (LDL), high density lipoprotein (HDL), fermentation, biomedical applications

## Abstract

Lovastatin is a potent hypercholesterolemic drug used for lowering blood cholesterol. Lovastatin acts by competitively inhibiting the enzyme, 3-hydroxy-3-methylglutaryl coenzyme A reductase involved in the biosynthesis of cholesterol. Commercially lovastatin is produced by a variety of filamentous fungi including *Penicillium* species, *Monascus ruber* and *Aspergillus terreus* as a secondary metabolite. Production of lovastatin by fermentation decreases the production cost compared to costs of chemical synthesis. In recent years, lovastatin has also been reported as a potential therapeutic agent for the treatment of various types of tumors and also play a tremendous role in the regulation of the inflammatory and immune response, coagulation process, bone turnover, neovascularization, vascular tone, and arterial pressure. This review deals with the structure, biosynthesis, various modes of fermentation and applications of lovastatin.

Lovastatin is an effective inhibitor of the enzyme hydroxymethylglutaryl coenzyme A (HMG-CoA) reductase (mevalonate: NADP1 oxidoreductase, EC 1.1.1.34) that catalyzes the reduction of HMG-CoA to mevalonate during synthesis of cholesterol^1^. When the lactone ring of lovastatin is in its open form, as it would be in the human liver, the structure bears a strong similarity to HMG-CoA. It has been shown that lovastatin and the other monacolins are very specific competitive inhibitors of the reductase^2^–^5^, which reduce serum cholesterol levels by blocking cholesterol biosynthesis as shown in the fig. 1. An attractive characteristic of these inhibitors is that they selectively reduce levels of low-density-lipoprotein (LDL), the bad lipoproteins. While levels of high-density lipoprotein (HDL, the good lipoproteins) remain unaffected, or in some cases, even increase. Lovastatin is currently made commercial by fermentation. However, other synthetic routes have also been considered. One of the reported routes is by a cell-free extraction in aqueous solution, where the lactone ring is in its open form^6^. Another method involves the use of silyl ethers^7^.

**Fig. 1:**
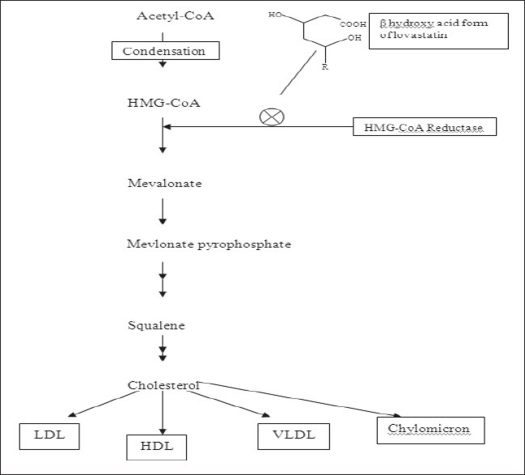
Role of lovastatin in inhibition of cholesterol synthesis The cellular and molecular mechanism of statins by considering the biosynthetic pathway of cholesterol. The main step leading to the reduction in cholesterol synthesis is the decrease in the precursor mevalonate by the inhibition of the HMG-CoA reductase. By inhibiting the HMG-CoA to mevalonate, the biosynthesis of two major downstream products of mevalonate, cholesterol production and synthesis of isoprenoids are influenced.

Lovastatin (or mevinolin, monacolin K, and Mevacor, Merck) contains a methylbutyric side chain (R1) and a 6-α methyl group (R2) as shown in fig. 2. The foremost query addressed was whether a reduction in plasma cholesterol level with lovastatin would be associated with a reduction in the whole body production rate of cholesterol or with the sizes of exchangeable body cholesterol pools as determined by the three-pool model of cholesterol turnover. The mean plasma cholesterol level decreased 19.4% (from 294 to 237 mg/dl), and low-density lipoprotein cholesterol decreased 23.8 % (from 210 to 159 mg/dl) with lovastatin therapy. Thus, HMG-CoA reductase inhibition by lovastatin at the therapeutic dose used here did not change the steady-state rate of whole-body cholesterol synthesis. Some of the study explained that lovastatin enhanced the healing rate and increased biomechanical properties of the bone at the fracture site. Lovastatin therapies have been shown to reduce cardiovascular events, including myocardial infarction, stroke and death, significantly, by altering vascular atherosclerosis development in patients with or without coronary artery disease symptoms^8^,^9^. Through the analysis of the inhibitory effect on HMG-CoA reductase, it is possible to highlight the influence of the obtained structures on biological activity. The stereochemistry of the side chain ester moiety is not important for inhibitory binding to HMG-CoA reductase, as the spatial requirements of the acyl moiety are compatible with compact, branched-chain aliphatic acyl groups, and additional branching at the α carbon of the acyl moiety increases potency.

**Fig. 2:**
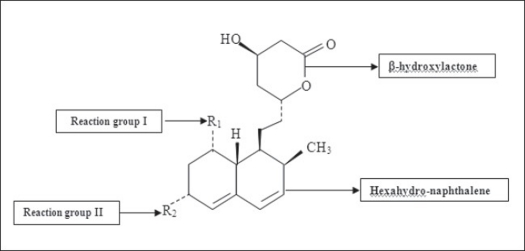
Structure of lovastatin All statins possess a common structure, a hexahydro-naphthalene system and a ß-hydroxylactone; their differences are due to side chains (R1) and methyl groups (R2) around the ring.

Various fungi such as *Aspergillus* (*A. terreus*) species, *Monascus* (*M. ruber, M. purpureus, M. pilosus, M. vitreus, M. pubigerus* and *M. anka*) species, *Paecilomyces viridis* and *Pencillium* (*P. citrinum*) species have been found to produce lovastatin^7^. Uncontrolled filamentous growth occurs when using rapidly metabolized substrates. The rapid increase in viscosity accompanied by filamentous growth greatly impedes oxygen transfer and this is said to explain the low titers of lovastatin. Fermentation-derived lovastatin is a precursor for simvastatin, a powerful semi-synthetic statin commercially available as Zocor™. Simvastatin is obtained via a selective enzymatic deacylation of lovastatin^8^. An alternative method for the lovastatin synthesis was semi-synthetic route where the synthesis occurs via the regioselective enzymatic esterification of 2-methylbutyric acid and a diol lactone precursor. The method has the potential advantage that different analogs of lovastatin can be synthesized from the same lactone precursor using different carboxylic acids. Lovastatin also inhibits tumor growth through the inhibition of non-sterol isoprenoid synthesis. Lovastatin (mevinolin) was the first hypocholesterolemic drug to be approved by Food and Drug administration (FDA), USA.

## VARIOUS MODES OF FERMENTATION

Submerged batch fermentation of lovastatin production was reported in various literatures and commercial production of lovastatin is based on *A. terreus* batch fermentation and most of the literature deals with this species^7^. *Aspergillus terreus* fermentations were typically carried out at 28° and pH 5.8-6.3. The dissolved oxygen level is controlled at 40% of air saturation. Batch fermentation generally runs for less than 10 days. In some cases, pelleted growth of *A. terreus* has yielded higher titers of lovastatin than obtained with filamentous growth. The composition of a fermentation medium influences the supply of nutrients and metabolism of cells in a bioreactor and therefore the productivity of a fermentation process depend on the culture medium used. Of the major culture nutrients, carbon and nitrogen sources generally play a dominant role in fermentation productivity because these nutrients are directly linked with the formation of biomass and metabolites^10^. Also, the nature and concentration of the carbon source can regulate secondary metabolism through phenomena such as catabolic repression. Response surface methodology (RSM) was also adapted in identifying the impact of the medium composition on lovastatin production with a high producing *A. terreus*. The effect of concentrations of several carbon and nitrogen sources were studied and a significant interactive effect of the medium constituents on lovastatin titers was observed^11^. The RSM technique was used to optimize the culture medium for producing lovastatin from *Monascus rubber*^12^. No report exists on any interactive effects of dissolved oxygen and the other nutrients on the production of lovastatin. Process optimization is a tedious process due to involvement of multivariable process parameters. Screening of important factors is initially carried out and the selected factors are then optimized by different techniques. RSM has some advantages that include less experiment numbers, suitability for multiple factor experiments, search for relativity between factors, and finding of the most suitable condition and forecast response^13^. Fed-batch fermentations of *A. terreus* have been investigated for producing lovastatin and are said to be superior to batch cultures, because of the feed-back inhibition of product to its own synthesis. Submerged fermentation processes for large-scale lovastatin production have been developed using *A. terreus* and other species^10^,^14^. Solid state fermentation uses economical substrates (agricultural residues), requires fewer processing and down-streaming stages, utilizes lesser power and generates lesser effluent. Moreover, SSF has higher product yield and offers better product stability. Because of the reasons, solid state fermentation was used mainly for the production of industrial enzymes but nowadays, it is also being exploited for the production of secondary metabolites. However, there is no discussion in the literature of how the concentration of lovastatin might affect its own synthesis by *A. terreus*. Product inhibition of fermentation is a crucial influence in many industrial processes, but has not been documented for lovastatin production^14^–^16^.

Biotransformation investigations of statins were carried out on ^14^C-labelled monacolin J and L, employing a strain of *Monascus ruber*, suggested that these compounds are precursors of lovastatin and consequently can be classified as isolated intermediate metabolites in the lovastatin biosynthetic pathway^17^. Subsequent experiments employing the cell-free extract of *M. ruber* and living cells of *Paecilomyces viridis* have demonstrated the transformation of monacolin J^6^. Moreover, a combination of physical techniques indicates monacolin M derivation from monacolin J, via a pathway that is quite distinct from that for the synthesis of lovastatin, the α-methylbutyryl ester of monacolin J^3^.

## BIOCHEMICAL PATHWAYS IN LOVASTATIN SYNTHESIS

From an overview of the early biogenetic studies carried out on the monacolins, it is possible to demonstrate that monacolin L is the first to be synthesized from nine molecules of acetate and is, in turn, converted to monacolin J by hydroxylation; monacolin K is then derived from monacolin J. The monacolin X, i.e., the α-methyl-β-ketobutyryl ester of monacolin J, is converted to lovastatin, while it is accumulated in cultures of mutant strains producing no detectable amounts of lovastatin^3^. The investigation of the biogenesis of lovastatin, carried out mainly in *Aspergillus terreus* strains employing labeled precursors indicated that the lovastatin biosynthetic pathway starts from acetate units (4- and 8-carbons long) linked to each other in head-to-tail fashion to form two polyketide chains. The methyl group present in some statins in the side chain or at C6 derives from methionine, as frequently occurs in fungal metabolism, and is inserted in the structure before the closure of the rings. Then the main chain is cyclized and in some statins esterified by a side chain at C8. The oxygen atoms present in the main chain are inserted later by aerobic oxidation using a deoxygenated precursor^8^,^18^. Studies on the ^13^C incorporation in lovastatin carried out with *Penicillium citrinum* and *M. ruber* strains indicated a similar pathway; enzymatic hydroxylation and subsequent esterification at C8 was also observed^17^.

More-recent investigations have studied the enzymatic kinetics together with gene regulation and expression involved in *A. terreus* lovastatin biosynthesis. The genetic research investigated the mechanisms involved in lovastatin biosynthesis, particularly with regard to the two polyketide chains. The results, including the characterization of *A. terreus* lovastatin-blocked mutants, showed that the multifunctional polyketide synthase system (PKSs) comprises a lovastatin nonaketide synthase (LNKS) involved in the cyclization of the main polyketide chain, to form the hexahydro naphthalene ring system, and a diketide synthase (LDKS) involved in the transfer of the methylbutyryl side chain to monacolin J. Study of the primary structure of the PKS that forms the lovastatin nonaketide provided new details of lovastatin biosynthesis. Other aspects of the biosynthesis of lovastatin related to PKSs have been investigated. The LNKS, product of *lovB* gene, interacts with *lovC* (a putative enoyl reductase), to catalyze the reactions in the first part of the biosynthetic pathway, leading to dihydromonacolin L. In the final step of the lovastatin pathway, the LDKS, made by *lovF*, interacts with *lovD* (transesterase enzyme) that catalyzes the attachment of the 2-methylbutyric acid to monacolin J, derived from monacolin L. Key features of genes encoding these enzymes and regulatory factors in lovastatin production in *A. terreus* have been elucidated^19^,^20^.

**Fig. 3:**
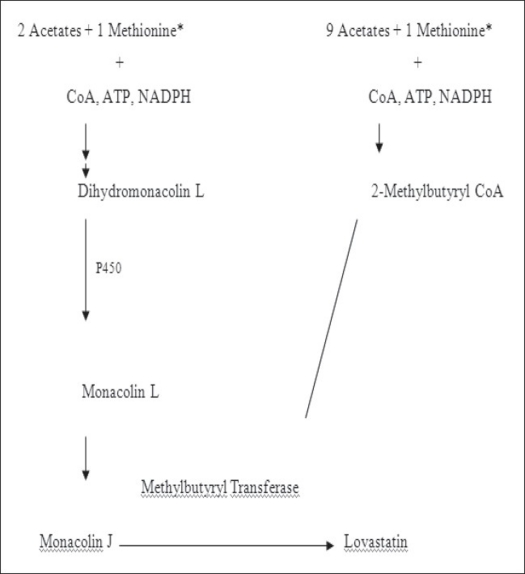
Biosynthesis of lovastatin The lovastatin biosynthetic pathway starts from acetate units linked to each other in head- to-tail fashion to form two polyketide chains. Monacolin L is first produced from acetate and methionine and then hydroxylated to monacolin J by a monooxygenase. Monacolin J is further converted to monacolin X by an esterification reaction, followed by transformation into the end-product lovastatin.

An intramolecular Diels-Alder endo closure of the hexaketide, to form a bicyclic system, with the same ring stereochemistry as dihydromonacolin L, catalyzed by LNKS purified from *A. nidulans* was recently demonstrated. Finally in a strain of *A. terreus*, in which the *lovC* gene has been disrupted, the post-PKS (post-polyketide synthase) steps involved in the biosynthesis of lovastatin were investigated. The results demonstrated that the role of the *lovC* protein is to ensure correct assembly of the nonaketide chain in lovastatin by the *lovB* protein. In contrast, the construction of the methylbutyrate side chain by the LDKS (*lovF* protein) does not require *lovC* protein. The study also demonstrated that the *lovC* protein has no detectable function in post-PKS processing of dihydromonacolin L. The recent advances in gene cloning have allowed the identification of most of the enzymes involved in lovastatin biosynthesis and have confirmed the biosynthetic pathways hypothesized in earlier investigations^21^,^22^. The detailed process of biosynthesis of lovastatin is shown in fig. 3.

## PRODUCTION OF LOVASTATIN

The investigations carried out since 1970s have indicated the possibility of obtaining a wide range of lovastatin as both the final products and intermediates of secondary microbial metabolism, or as products of biotransformation process. Large-scale processes have been developed only for a few of the lovastatin described in the literature. For other molecules research is still ongoing and therefore greatly susceptible to future development. Lovastatin was the first statin to be approved by the Food and Drug Administration, United States (1987) and made available on the pharmaceutical market as an anticholesterolemic drug^23^. However, mevastatin was the first statin discovered. Lovastatin (named mevinolin) was later obtained from a strain isolated from soil and classified as *A. terreus* at the CIBE Laboratories in Madrid (Spain)^8^ and it is also obtained from *M. ruber* (named monacolin K). A few years later, lovastatin was also obtained from 17 strains of different species of 124 tested strains of the genus *Monascus*, in particular *M. ruber, M. purpureus, M. pilosus, M. vitreus*, and *M. pubigerus*^24^. The genus *Monascus*, particularly the species *M. anka* and *M. purpureus*, is traditionally employed in Asian countries as “red koji” for fermented food (red yeast rice) and beverage production, as well as for red dye. Lovastatin-producing strains are, instead, generally poor producers of red pigments and exhibit an optimum temperature for lovastatin production of around 25°. Lovastatin productivity failed and no increase in red pigments was observed when the temperature range for koji production (30-37°) was employed^24^,^25^. Studies on the synthesis and characterization of the lovastatin-related compounds indicated that several monacolins were obtainable, mostly from *Monascus* strains. Monacolin J and L were isolated and characterized from cultures of an *M. ruber* strain^3^,^4^,^26^. In 1985, Endo has reported dihydromonacolin L and monacolin X production and activity from a mutant strain of *M. ruber*^5^. A series of statins were also obtained by chemical modification of the C8 side chain in the lovastatin molecule and a systematic evaluation of the structure-activity relationships of the obtained compounds was also carried out. One of the obtained molecules, simvastatin was found to be a semi-synthetic molecule with practical applications^27^,^28^. Biotransformation studies carried out in the lovastatin to obtain a new and powerful statins^3^,^17^,^29^.

The industrial process for the production of lovastatin was set up in 1980 using an *A. terreus* strain (Mevacor, Merck). The process development involved the analysis of different fermentation parameters such as culture homogeneity, effect of various carbon sources, pH, aeration, and agitator design. Producer strain reisolation together with pH control and slow use of the carbon source, in particular glycerol, yielded a fivefold increase (about 200 U/l, arbitrary units) with respect to the initial lovastatin productivity. Scaling-up of the process from an 800 l to a 19,000 l scale revealed that oxygen transfer, related to high viscosity of the fermentation broth, is a serious limiting factor in lovastatin productivity. This limitation was overcome by setting up a more-efficient impeller with increased hydrodynamic thrust and a reduction of power requirement, 66% of that of the Rushton standard turbine^30^.

Metkinen group (The original lovastatin producer) increased the lovastatin production by *A. terreus* ATCC 20542 strains to reach 7-8 g/l, using mutagenesis procedures and experience acquired in the development program of process improvement. Biocon (Biocon India, Bangalore, India) is one of the companies that have obtained US FDA approval for lovastatin production (January 2001), and patented in June 2001. The company's lovastatin process is based on a proprietary fermentation technology, the Plafractor, a large-scale solid-matrix bioreactor. This new bioreactor has the advantages of solid substrate and submerged fermentation, and allows a reduction of downstream processing problems during product extraction^31^.

The production of biomass and lovastatin by spore-initiated submerged fermentations of *Aspergillus terreus* ATCC 20542 was studied and shown that the production depends on the age of the spores used for inoculation and the lovastatin titer was found to be 186.5±20.1 mg/l for a spore age of 16 days. The time to sporulation on surface cultures was sensitive to the light exposure history of the fungus and the spore inoculation concentration levels^32^.

The optimized fermentation conditions raised the lovastatin titer by four-fold compared with the worst-case scenario within the range of factors and this study was also investigated that the culture medium had excess carbon but limiting amounts of nitrogen source for the better productivity. This study used statistical analysis in documenting the interactions between oxygen supply and nutrient concentrations in lovastatin production. The Box–Behnken design was used to identify the oxygen content in the gas phase as the principal factor influencing the production of lovastatin. Both a limitation and excess of oxygen reduced lovastatin titers^14^.

In batch fermentation, lovastatin biosynthesis with *Aspergillus terreus* reached 160 U/l in 161 h at pH 6.8 and a dissolved O_2_ tension maintained at 70% and the yield of lovastatin produced in repeated fed batch fermentations was increased by 37% though this took over twice as long as in the batch fermentation. Accumulation of lovastatin suppresses its own synthesis in the microfungus *Aspergillus terreus* through a feed back regulatory mechanism and hence the product was removed continuously from the production medium. Submerged cultivation of a high yielding strain of *Aspergillus terreus* DRCC 122 for the production of lovastatin in the batch process has limited success with a maximum titre of 1270 mg/l in 288 h and an overall volumetric productivity of 4.41 mg/l h in a 1000 l bioreactor. A cost effective repeated fed-batch process with maltodextrin and corn steep liquor feed as carbon and nitrogen sources, respectively, showed a significant increase in lovastatin yield. The final titre was 2200 mg/l in 288 h of fermentation, with overall volumetric productivity of 7.64 mg/l h, showing an increase of 73% over the batch process. The maximum specific oxygen uptake rate (*Q*O_2_) and volumetric mass transfer coefficient (K_L_a) were 0.24 m mole O_2_ per g dry cell wt. per h and 280 per h, respectively, in fed- batch process. Homogenity and stability of high producing strain of *Aspergillus terreus*, the rate of utilization of the carbon source, pH control and high level of dissolved O_2_ tension (DOT) are of essential importance for high lovastatin production. In carbon source, the glycerol improved lovastatin production by 30% than the glucose^10^,^33^.

Among several organic and inorganic defined nitrogen sources metabolized by *A. terreus*, glutamate and histidine gave the highest lovastatin biosynthesis level. For cultures on glucose and glutamate, lovastatin synthesis initiated when glucose consumption leveled off. When *A. terreus* was grown on lactose, lovastatin production initiated in the presence of residual lactose. Experimental results showed that carbon source starvation is required in addition to relief of glucose repression, while glutamate did not repress biosynthesis^12^.

A lovastatin-hyperproducing culture of *Aspergillus terreus* has shown to produce several co-metabolites extracted from whole broth. The predominant co-metabolite was the benzophenone, sulochrin, reported to arise from a polyketide biosynthetic pathway. This compound was targeted for suppression by classical mutagenesis and screening and this gives raise to increased production of lovastatin than its co-metabolites^34^. Five nutritional parameters were screened using Plackett–Burman experimental design and were optimized by Box–Behnken factorial design of response surface methodology for lovastatin production in shake flask cultures by *M. purpureus* MTCC 369. Maximum lovastatin production of 351 mg/l were predicted in medium containing 29.59 g/l dextrose, 3.86 g/l NH_4_Cl, 1.73 g/l KH_2_PO_4_, 0.86 g/l MgSO_4_·7H_2_O, and 0.19 g/l MnSO_4_·H_2_O using response surface methodology^12^.

The production of lovastatin and microbial biomass by *Aspergillus terreus* ATCC 20542, were studied and the production was influenced by the type of the carbon source (lactose, glycerol, and fructose) and the nitrogen source (yeast extract, corn steep liquor and soybean meal) used and the C:N mass ratio in the medium. Use of a slowly metabolized carbon source (lactose) in combination with either soybean meal or yeast extract under *N*-limited conditions gave the highest titers and specific productivity (0.1 mg/g h) of lovastatin. The maximum value of the lovastatin yield coefficient on biomass was 30 mg/g using the lactose/soybean meal and lactose/yeast extract media. The optimal initial C: N mass ratio for attaining high productivity of lovastatin was 40. The behavior of the fermentation was not affected by the method of inoculation (fungal spores or hyphae) used, but the use of spores gave a more consistent inoculum in the different runs^11^.

Product quality and high yields of secondary metabolites are the main goals for the commercial success of a fermentation process. A novel approach based on the decision-tree algorithm to determine the key variables correlated with the process outcome and on DOSY-NMR to identify both co-metabolites and impurities was used which improves fermentation systems and speeds up bioprocess development. The approach has been validated in the case of lovastatin production from *Aspergillus terreus* and showed that the NMR spectroscopy increased speed and productivity of a fermentation process by reducing large scale verification experiments and cost of purification steps; moreover, this approach allows to monitor and guarantee the product chemical validation suitable for pharmacological uses^35^.

A two-stage feeding strategy was shown to improve the rate of production of lovastatin by more than 50% when compared with conventional batch fermentation by *Aspergillus terreus*. The feeding strategy consisted of an initial batch/fed-batch phase and a semi-continuous culture dilution phase with retention of pelleted biomass in a slurry bubble column reactor. The batch phase served only to build up the biomass for producing lovastatin, a secondary metabolite that inhibits its own synthesis in the producing microfungus. The semi-continuous dilution phase provided nutrients to sustain the fungus, but prevented biomass growth by limiting the supply of essential nitrogen. The preferred pelleted growth morphology that favors lovastatin synthesis was readily obtained and maintained in the bubble column reactor. In contrast, stirred tank fermentation had a substantially lower production of lovastatin because mechanical agitation damaged the fungal pellets^36^.

## BIOMEDICAL APPLICATIONS OF LOVASTATIN

### Coronary heart disease (CHD):

Statins are the treatment of choice for the management of hypercholesterolaemia because of their proven efficacy and safety profile and they can exert antiatherosclerotic effects independently of their hypolipidemic action. Since the lovastatin metabolism generated a series of isoprenoids vital for different cellular functions, from cholesterol synthesis to the control of cell growth and differentiation, HMG-CoA reductase inhibition has beneficial pleiotropic effects^37^. Consequently, lovastatin reduce significantly the incidence of coronary events, both in primary and secondary prevention, being the most efficient hypolipidemic compounds that have reduced the rate of mortality in coronary patients. Lovastatin also have an increasing role in managing cardiovascular risk in patients with relatively normal levels of plasma cholesterol. Large-scale clinical trials have demonstrated that the statins substantially reduce cardiovascular-related morbidity and mortality in patients with and without existing CHD. Observational studies have demonstrated an increased risk of ischemic stroke at high cholesterol levels and an increased risk of haemorrhagic stroke at low cholesterol levels. It is suggested that low cholesterol may predispose to haemorrhagic stroke by contributing to a weakening of the endothelium of small cerebral arteries. Many statin trials have focused on coronary events and total mortality. Lovastatin basically improves the endothelial function, modulates inflammatory responses, maintain plaque stability and prevent thrombus formation, with which all sorts artery related diseases could be cured and it has been suggested that the consequence of the shrinkage of the lipid core of the atherosclerotic plaque, avoiding plaque rupture that would otherwise trigger intramural hemorrhage and intraluminal thrombosis^38^,^39^.

### Cholesterol lowering actions:

Cholesterol is generally synthesized in the liver, and statins work primarily by inhibiting an enzyme involved in its synthesis a complex. 3-hydroxy-3-methyglutaryl coenzyme A is converted into mevalonate, a precursor of cholesterol, in the presence of the enzyme HMG CoA reductase^8^. Lovastatin is the hydrophobic ring structure that was covalently linked to the substrate analogue which involved in binding to the reductase enzyme and inhibiting the cholesterol synthesis. This rate-limiting step in cholesterol biosynthesis is blocked by statins. This also reduce the LDL level which cause arthrosclerosis and increase the level of HDL which acts as good cholesterol and it avoids the lesion formation in the artery that leads to narrow down the blood circulation through the arteries but the mechanism was unknown^9^.

### Drugs for Alzheimer's disease (AD):

Lovastatin treatment was observed to reduce the prevalence of AD in patients suffering from hypercholesterolaemia. Many of the known risk factors for AD were associated with cholesterol metabolism. Interestingly, it seems as if higher doses of lovastatin, that is inhibitors of the cholesterol biosynthesis by blocking formation of mevalonate, might lower the progression of AD. The mechanisms, however, by which lovastatin or cholesterol levels exert their influence are unknown. The alternative processing of the amyloid-precursor protein (APP) in the brain of AD patients leads to the production of the neurotoxic amyloid-beta protein (Ab), a causative factor for AD pathology^40^. These findings led to prospective clinical trials of cholesterol-lowering statins in AD patients, even if many studies do not support elevated cholesterol levels in serum and brain as a risk factor for Alzheimer's disease. Thus, upto date there is insufficient evidence to suggest the use of lovastatin for treatment in patients with AD. Several studies demonstrated that the cleavage of APP can be modulated by altering membrane cholesterol levels *in vitro*^41^.

### Lovastatin in renal disease treatment:

The important advances have been used in the treatment of patients with progressive renal disease. The inhibitors of HMG-CoA reductase can provide protection against kidney diseases characterized by inflammation and/or enhanced proliferation of epithelial cells occurring in rapidly progressive glomerulonephritis, or by increased proliferation of mesangial cells occurring in IgA nephropathy. The mechanisms underlying the action of statins are not yet well understood, although recent data in the literature indicate that they can directly affect the proliferation/apoptosis balance, the down regulation of inflammatory chemokines, and the cytogenic messages mediated by the GTPases Ras superfamily. Lovastatin may directly influence intracellular signaling pathways involved in the prenylation of low molecular weight proteins that play a crucial role in cell signal transduction and cell activation. As far as kidney diseases are concerned, lovastatin therapy has been shown to prevent creatinine clearance decline and to slow renal function loss, particularly in case of proteinuria, and its favorable effect may depend only partially on the attenuation of hyperlipidemia^42^.

### Lovastatin and cancer:

In primary cultures of human glioblastoma cells, inhibition of Ras farnesylation by lovastatin is associated with reduction of proliferation and migration. So the proliferations of the cancer cells were inhibited by lovastatin. However, the inhibition of cell growth by lovastatin may be independent of Ras function. In C6 glioma cells treated with lovastatin, free geranylgeraniol overcomes the arrest of cell proliferation, whereas the rescue effect was significantly lower with farnesol. Two recent case control studies, involving over 10,000 individuals, have looked for changes in the rates of cancer at specific sites, but failed to demonstrate a clear association with statin use^43^,^44^.

### Lovastatin used in bone fraction treatment:

One of the recent trends is that the treatment of bone fracture is by lovastatin^16^. Lovastatin stimulate bone formation *in vitro* and *in vivo* and, when given in large doses or by prolonged infusions, stimulate biomechanical strength of murine long bones with healing fractures. However, administration of lovastatin by large oral doses or prolonged infusions to a fracture site is not a feasible therapeutic approach to hasten healing of human fractures. Lovastatin in biodegradable polymer nanobeads of poly (lactic-co-glycolide acid) to determine if lovastatin delivered in low doses in nanoparticles of a therapeutically acceptable scaffold could increase rates of healing in a standard preclinical model of femoral fracture. Some preclinical studies were suggested that lovastatin administered in a nanobead preparation may be therapeutically useful in hastening repair of human fractures^16^. Garrett *et al*^16^ found that these nanobeads: 1. Stimulated bone formation in vitro at 5 μg/mL, 2. Increased rates of healing in femoral fractures when administered as a single injection into the fracture site, and 3. Decreased cortical fracture gap at 4 weeks as assessed by microcomputed tomography. These preclinical studies were suggested that lovastatin administered in a nanobead preparation may be therapeutically useful in hastening repair of human fractures^16^.

### Other applications:

Lovastatin is also used for the inhibition of the induction of inducible nitric oxide synthase and proinflammatory cytokines in rat astrocytes, microglia and macrophages and to repress MHC-II mediated T-cell activation. Moreover, lovastatin treatment decreased neuroinflammatory activity and clinical signs in experimental allergic encephalomyelitis, an animal model for multiple sclerosis (MS)^45^,^46^. In the last few years many studies have demonstrated that statins, in addition to their lipid lowering effects, have antiinflammatory and immunomodulatory properties. These properties of statins have suggested that they could have beneficial effects in immune mediated neurological disorders. Lovastatin therapy can significantly reduce morbidity and mortality in diabetics.

## CONCLUSIONS

Lovastatin inhibit HMG-CoA reductase competitively; decreases LDL level more than other cholesterol-lowering drugs, and lower triglycerides level in hypertriglyceridemic patients. Lovastatin have antiatherosclerotic effects, which correlate positively with the percent decrease in LDL cholesterol. In addition, lovastatin can exert antiatherosclerotic effects independently of their hypolipidemic action. Because the mevalonate metabolism creates a series of vital isoprenoids for different cellular functions, from cholesterol synthesis to the control of cell growth and differentiation, HMG-CoA reductase inhibition has beneficial pleiotropic effects. Lovastatin have become the therapy of interest for the treatment of many dyslipidaemias in the patients with Alzheimer's disease, renal disease treatment, cancer treatment, bone fracture treatment and used as immunosuppressant. Although lovastatin share a common mechanism of action, there are differences in their relative efficacy for improving the lipid profile, as well as in their chemistry and pharmacokinetics. Lovastatin is well tolerated and have an excellent safety record. Consideration of these differences should help to provide a coherent basis for the safe and effective use of the current and budding lovastatin in clinical practice.
